# Image-guided navigation for locally advanced primary and locally recurrent rectal cancer: evaluation of its early cost-effectiveness

**DOI:** 10.1186/s12885-022-09561-w

**Published:** 2022-05-06

**Authors:** Melanie Lindenberg, Astrid Kramer, Esther Kok, Valesca Retèl, Geerard Beets, Theo Ruers, Wim van Harten

**Affiliations:** 1 Health Technology and Services Research, University of Twente, Enschede, The Netherlands; 2 Division of Psychosocial Research and Epidemiology Netherlands Cancer Institute, Antoni van Leeuwenhoek, Amsterdam, The Netherlands; 3 Department of Surgical Oncology, Netherlands Cancer Institute – Antoni van Leeuwenhoek, Amsterdam, The Netherlands; 4 Faculty TNW, Group Nanobiophysics, Twente University, Enschede, The Netherlands

**Keywords:** Early cost-effectiveness analysis, Navigation technology, Surgery, Early health technology assessment, Locally advanced rectal cancer, Local recurrent rectal cancer

## Abstract

**Background:**

A first pilot study showed that an image-guided navigation system could improve resection margin rates in locally advanced (LARC) and locally recurrent rectal cancer (LRRC) patients. Incremental surgical innovation is often implemented without reimbursement consequences, health economic aspects should however also be taken into account. This study evaluates the early cost-effectiveness of navigated surgery compared to standard surgery in LARC and LRRC.

**Methods:**

A Markov decision model was constructed to estimate the expected costs and outcomes for navigated and standard surgery. The input parameters were based on pilot data from a prospective (navigation cohort *n* = 33) and retrospective (control group *n* = 142) data. Utility values were measured in a comparable group (*n* = 63) through the EQ5D-5L. Additionally, sensitivity and value of information analyses were performed.

**Results:**

Based on this early evaluation, navigated surgery showed incremental costs of €3141 and €2896 in LARC and LRRC. In LARC, navigated surgery resulted in 2.05 Quality-Adjusted Life Years (QALYs) vs 2.02 QALYs for standard surgery. For LRRC, we found 1.73 vs 1.67 QALYs respectively. This showed an Incremental Cost-Effectiveness Ratio (ICER) of €136.604 for LARC and €52.510 for LRRC per QALY gained. In scenario analyses, optimal utilization rates of the navigation technology lowered the ICER to €61.817 and €21.334 for LARC and LRRC. The ICERs of both indications were most sensitive to uncertainty surrounding the risk of progression in the first year after surgery, the risk of having a positive surgical margin, and the costs of the navigation system.

**Conclusion:**

Adding navigation system use is expected to be cost-effective in LRRC and has the potential to become cost-effective in LARC. To increase the probability of being cost-effective, it is crucial to optimize efficient use of both the hybrid OR and the navigation system and identify subgroups where navigation is expected to show higher effectiveness.

**Supplementary Information:**

The online version contains supplementary material available at 10.1186/s12885-022-09561-w.

## Background

Rectal cancer is mainly treated by surgical resection, often complemented with pre- and/or postoperative (chemo) radiotherapy in stage II-IV tumors [1–3], showing a 5-year survival rate of ~ 45% for stage III and ~ 20% for stage IV tumors [4]. Surgical resection of both locally advanced (LARC) and locally recurrent rectal cancer (LRRC) requires special consideration because (1) the disruption of normal anatomical planes and (2) radiotherapy-induced fibrosis can lead to a higher risk of a tumor positive involved circumferential resection margin [1, 5]. In this setting, LARC was defined as T3 or T4 tumors extending close to (< 2 mm) or invading the mesorectal fascia, as shown on rectal magnetic resonance imaging. LRRC was defined as rectal cancer that recurred in the pelvic area after earlier treatment. In 10–15% of rectal cancer patients, positive surgical margins are found [6, 7] which negatively affects the prognosis [8–10]. Local recurrence can cause debilitating symptoms, and often requires additional treatment, such as chemoradiotherapy and radiotherapy. Optimizing surgical practice and decreasing the risk of positive resection margins is therefore of great clinical and financial importance.

Multiple technologies have emerged to improve the quality of surgery and surgical outcomes [11]. The Netherlands Cancer Institute (NKI-AVL) has developed an image-guided navigation system to improve tumor localization during the operative procedure and prevent damage to surrounding vital structures [12]. Recently, this navigation system has been evaluated in the first series of LARC and LRRC patients, showing substantially improved negative surgical margin rates compared to standard surgery in a historical control group [12]. Since the use of a navigation system is associated with extra costs (e.g. due to extra imaging, the navigation system, and personnel), and hospital budgets are limited, new surgical technologies have to prove themselves in terms of cost-effectiveness to have a chance of reimbursement.

To evaluate the potential value of this navigation system, to inform policymakers, and to guide subsequent decisions on further research and development [13], early cost-effectiveness analyses can be performed. This study evaluates the early cost-effectiveness of the image-guided navigation system used during surgery for LARC and LRRC patients compared to standard surgery based on the first clinical data sampled in the Netherlands Cancer Institute [12].

## Methods

### Study design and model structure

**Fig. 1:**
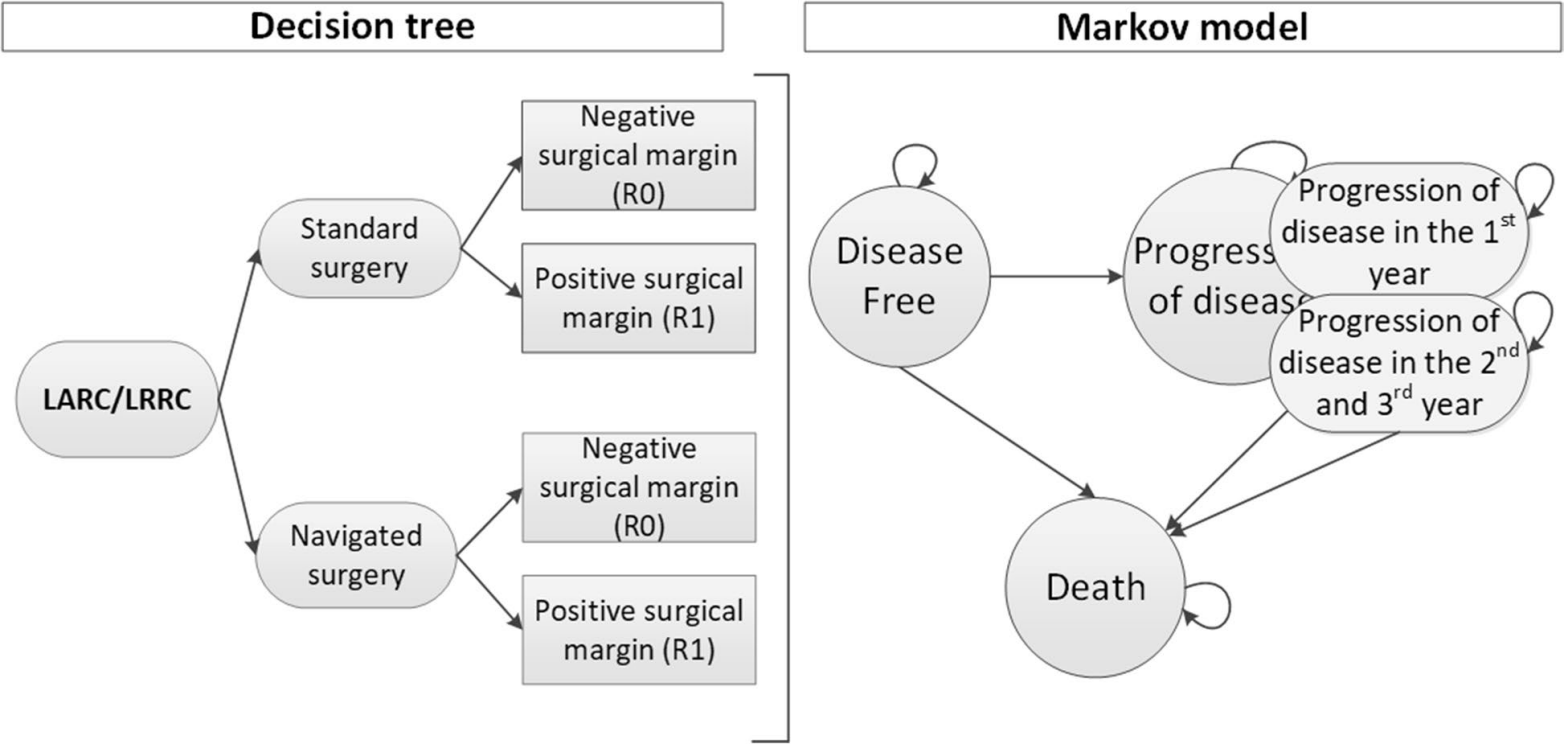
Overview of the model. On the left, the decision tree is visualized in which the margin status after navigated and standard surgery is incorporated. On the right, the Markov model is shown which is used to model the costs and effects after having a negative or positive surgical margin. It also shows the tunnel states used to incorporate time effects on the transition from progression to death due to progression

To evaluate the early cost-effectiveness of navigated surgery we used a combination of a decision tree and a Markov model. The decision tree showed the possibility of having a positive (R1) or negative (R0) resection margin after standard and navigated surgery [12]. The Markov model comprised the mutually exclusive health states: “disease-free”, “progression of disease” and the absorbing state “death” (Fig. 1). Whether a patient moves is partly explained by the outcome of the decision tree (R1 or R0). In the Markov model, all patients start in “disease free” and could either remain in “disease free” or transfer to “progression of the disease” or “death”. Since the course of disease for LARC and LRRC is different, two separate models were constructed with a similar design. The time horizon was set at 3 years because most recurrences develop in the first 3 years after (curative) resection [14]. Besides, recent literature reported a median survival time of 37 [15] and 30 months [16] for LARC and LRRC, respectively. A cycle time of 3 months was chosen according to guidelines for follow-up visits [17]. The early cost-effectiveness analysis was performed from a Dutch healthcare perspective, using the Dutch guideline for health economic costing studies [18]. This means that we evaluate all relevant costs and effects part of the healthcare system, e.g. productivity losses or travel expenses of patients were not included. The primary outcome of this analysis is the incremental cost-effectiveness ratio (ICER).

### Standard and navigated surgery

Standard treatment of LARC and LRRC consists of a rectal resection performed with an Abdomioperineal Resection (APR) or a Low Anterior Resection (LAR), with or without resection of the surrounding organs (exenterative procedures, sacral bone etc.) and intra-operative radiotherapy, depending on the patient and tumor characteristics (e.g. tumor location, previous surgeries, etc.). Procedures can be performed open or laparoscopically.

The addition of the navigation system for rectal surgery in patients with LARC or LRRC changed the regular workflow before and during surgery. One day before surgery, a multiphase contrast- enhanced CT scan (with early arterial and excretion phase) was acquired. Based on this preoperative imaging a digital 3-dimensial anatomical model was made, including the most important anatomical structures (blood vessels, ureters, bones and targets). Before surgery, in a hybrid operating room, three patient trackers (electromagnetic) were taped to the skin of the patient, and a cone-beam CT scan was performed. The acquired intraoperative images were matched with the preoperative images and the 3-dimensial anatomical model. During surgery, the patient lies on a specific imaging bed including an electromagnetic field generator. The location of the patient trackers was matched with the preoperative imaging and 3-dimensional anatomical model. By using an electromagnetic pointer, the surgeon could navigate towards the tumor in the 3D anatomical model on a separate screen. A more detailed description of the navigation system can be found in the article of Nijkamp et al., 2018 [19].

### Input parameters

The input parameters are presented in Tables 1, 2, 3. Supplement 1 shows a schematic overview of the data sources used for the input parameters.

**Table 1:** Input parameters for the decision tree and the Markov model on clinical effectiveness

**The observed number of patients**	**LARC**		**LRRC**			**Source**
**Having a negative surgical margin (R0)**						
After navigated surgery	13 (*n* = 14)		15 (*n* = 19)			[A]
After standard surgery	85 (*n* = 101)		20 (*n* = 41)			[B]
**Having progression per year**						
after R0 1st year	29 (*n* = 85)		9 (*n* = 20)^c^			[B]
after R0 2nd (for LRRC: and 3rd) year	9 (*n* = 85)		4 (*n* = 20)^c^			[B]
after R0 3rd year^a^	4^a^(*n* = 85)		–			[B]
after R1 1st year	11 (*n* = 16)		11 (*n* = 20)			[B]
after R1 2nd and 3rd year	1 (*n* = 16)		3 (*n* = 20)			[B]
**Died due to CRC after progression; over a timeframe of 3 years**						
after progression in the 1st year	25 (*n* = 41)^d^		13 (*n* = 20)			[B]
after progression in the 2nd and 3rd year	2 (*n* = 13)^d^		3 (*n* = 7)			[B]
**Parameters used in the decision model**	**LARC**		**LRRC**		**Distribution**	**Source**
	**Mean**	**SE**	**Mean**	**SE**		
**Negative surgical margin rate (R0)**						
Navigated surgery	0.93	0.0665	0.79	0.0911	Beta	[A]
Standard surgery	0.84	0.0362	0.49	0.0771	Beta	[B]
**Transition probability for Disease-free to Progression after a negative surgical margin (R0)**						
from DF to PD in the 1st year	0.103	0.0328	0.159	0.0798	Beta	[B]
from DF to PD in the 2nd year	0.047	0.0229	0.100	0.0656	Beta	[B]
from DF to PD in the 3rd year	0.013	0.0121	0.100^b^	0.0656	Beta	[B]
**Transition probability for Disease-free to Progression after a positive surgical margin (R1)**						
from DF to PD in the 1st year	0.252	0.105	0.252	0.0926	Beta	[B]
from DF to PD in the 2nd year	0.0275	0.0397	0.159	0.0780	Beta	[B]
from DF to PD in the 3rd year	0.0275^b^	0.0397	0.159^b^	0.0780	Beta	[B]
**Transition probability for Progressive Disease to Death**						
from PD in the 1st year to Death	0.090	0.0665	0.135	0.0745	Beta	[B]
from PD in the 2nd and 3rd year to Death	0.030	0.0362	0.089	0.1007	Beta	[B]
**Transition probability for Disease-free to Death**	0.0028	–	0.0044	–	–	[20] background mortality

*SE* Standard error, *DF* Disease Free, *PD* progression of disease, *CRC* ColoRectal Cancer; [A] Prospective data collection within the navigated group at the NKI-AVL [12]; [B] Retrospective data collection within the control group at the NKI-AVL [12]

^a^Only in the LARC group, among patients showing a negative surgical margin enough events were found in both the 2nd and 3rd year to calculate probabilities for both years. In the other groups, we found limited events and decided to calculate a combined probability for the 2nd and 3rd year

^b^shows the transitions that were similar for the 2nd and 3rd years. This probability was based on the sum of events occurring in the 2nd and 3rd years

^c^1 of the LRRC patients received two surgeries and were both included in the analysis by Kok et al. For evaluating progression of disease this does not make sense, therefore this patient was excluded. Therefore the sum is 40 instead of 41

^d^The total number of patients having progression in the 1st, and 2nd and 3rd year is different from the number presented between brackets in the lines for died due to progression. After R1 in the 1st year, all 12 events occurred in the 1st year and none in the 2nd and 3rd year. To incorporate uncertainty surrounding the chance on having progression in the 2nd and 3rd year we moved 1 event to the second year to calculate the transitions from disease-free to progression. Therefore, the number of patients progressed in the row for patients died due to progression shows one person more for the 1st year, and one person less for the 2nd and 3rd year

**Table 2:** Intervention and state costs and utilities used in the Markov model

**State costs**	**LARC**	**SE**	**LRRC**	**SE**	**Distribution**	**Source**
Disease-free	€ 492	€ 63	€ 492	€ 63	Gamma	Expert
Transition from DF to PD	€ 14.883	€ 1.898	€ 13.107	€ 1.672	Gamma	Expert
Progressive disease	€ 585	€ 75	€ 585	€ 75	Gamma	Expert
**Intervention costs**	**Combined LARC & LRRC**			**SE**	**Distribution**	**Source**
Surgery	€10.970			€1.399	Gamma	[21]
Addition of navigation	€3.388			€432	Gamma	Expert
Developing 3D model and preoperative CT scan	€ 269					[22–24]
Additional personnel during OR	€ 197					[22, 24]
Navigation system	€ 2.745					List prices; expert
Overhead	€ 177					[22]
**Utilities**	**Combined LARC & LRRC**			**SE**	**Distribution**	**Source**
First cycle	0.70 (n = 63)			0.029	Beta	[C] (1mo survey)
Disease free (subsequent cycles)	0.85 (*n* = 44)			0.022	Beta	[C] (6mo survey)
Progressive disease (subsequent cycles)	0.77 (n = 14)			0.050	Beta	[C] (6mo survey)

*SE* Standard error, *mo* month, [C] Prospective observational cohort study

#### Clinical effectiveness

The effectiveness of navigated surgery compared to standard surgery in terms of R0 or R1 were obtained from the patient population of the study of Kok et al. [12]. They prospectively included 33 patients who received navigated surgery for either LARC (*n* = 14) or LRRC (*n* = 19) between 2016 and 2019 in the Netherlands Cancer Institute (NKI-AVL). As a control group, Kok et al. included 142 patients having standard surgery for LARC (*n* = 101) and LRRC (*n* = 41) as a retrospective cohort. These patients had a similar indication and type of surgery at the NKI-AVL [12]. Supplement 2 shows the characteristics of these patient populations (prospective and retrospective group) [12]. The Institutional Review Board of the NKI-AVL approved data extraction for the included patients.

Among LARC patients, 93% R0 resections were achieved after navigated- and 84% after standard surgery. Among LRRC patients, 79% had an R0 resection after navigated- and 49% after standard surgery [12]. These values were incorporated in the decision tree.

To calculate the transitions between the health states in the Markov model, progression of disease was evaluated in the retrospective control group (*n* = 142). Based on literature, we assumed that (1) progression of disease was affected by the resection margin status [5, 26] and (2) that death due to colorectal cancer (CRC) was affected by progression status. Information on progression of disease stratified by margin status and mortality data stratified by progression status were retrieved from medical records. Progression of disease was defined as “local recurrence or distant metastasis after surgery”, as the sample size was too small to stratify for local and distant recurrence. Among these patients, some had limited metastatic disease prior to surgery (e.g. liver metastasis). To prevent overestimating the risk of progression in the whole population, these patients were incorporated in the progression of disease state in the first cycle after surgery.

**Fig. 2:**
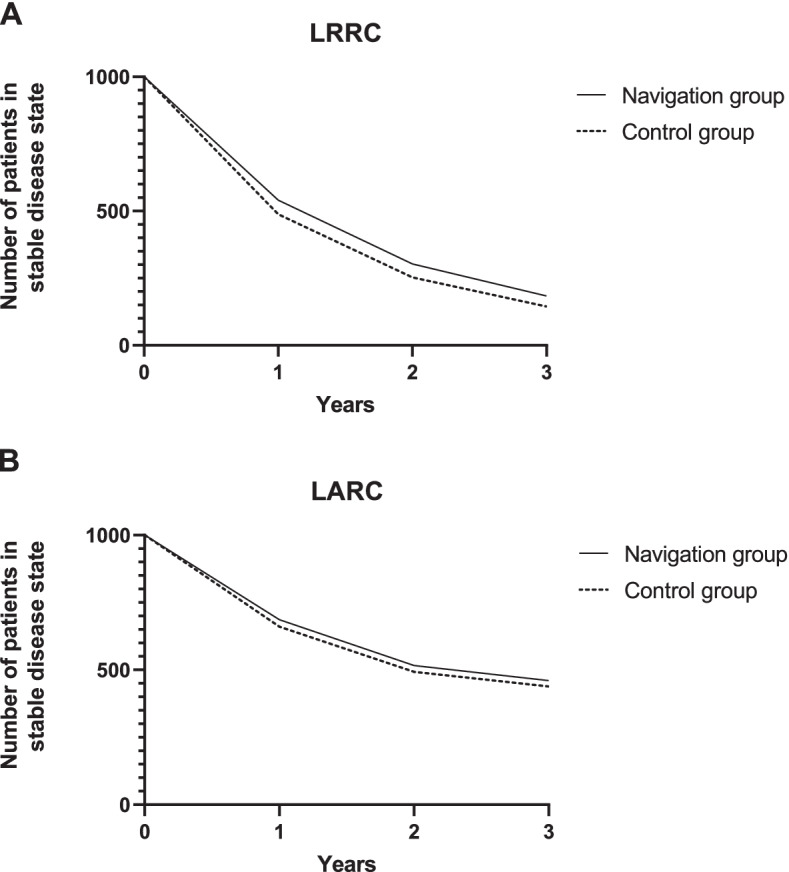
Graphical representation of patients in the stable disease state over time. These graphs show the number of patients in the model (cohort of 1000 patients) that stay in the stable disease state over time for the navigated and standard surgery group. **A** shows the patient flow for LRRC and **B** shows the patient flow for LARC

The probabilities to experience events (progression or death) per 3 months were calculated linearly using the number of events and the total number of patients at risk with the following formula: 1-exp(−r*t). Where ‘r’ stands for the rate per 3 months calculated by -(ln (1-observed chance)/time of the observation), and ‘t’ stands for the time [27]. To incorporate time or disease history in the model, two tunnel states were incorporated in the model: “1^st^-year progression of disease” and “2^nd^ or 3^rd^-year progression of disease” [28, 29]. The risk of dying due to progression within 3 years was evaluated separately for patients having progression in the first year and separately for patients having progression in the second and third year. The second and third year were combined because of a limited number of cases. Mortality due to all causes was based on data from the Dutch Central Bureau for Statistics, mirroring the average age of the two patient populations (LARC:60 years and LRRC:65 years) [12, 20]. All observed events and transition probabilities incorporated in the Markov model are listed in Table 1. Figure 2 shows the number of patients in the stable disease state over time for both interventions and patient groups.

#### Health-related quality of life

Since no representative Quality of Life (QoL) data was available in literature [30], we used data from 63 patients of an ongoing prospective cohort study of patients with colorectal cancer who undergo standard and navigated surgery within the NKI-AVL with similar inclusion criteria as the patients enrolled in the study of Kok et al. [12]. The clinical characteristics of these patients are presented in Supplement 3. Based on these clinical characteristics, the group was judged sufficiently comparable to the control group to be used in our analysis.

Utilities were measured among these patients using the EQ5D-5L [31] to incorporate Quality Adjusted Life Years (QALYs) in the model. Utilities are values between 0 and 1 where a higher value indicates a better health status. Patients in the ongoing cohort study complete questionnaires before surgery and after 1, 3, 6, 12, and 24 months. The utility value for the first cycle in the model was based on the first-month questionnaire, incorporating the impact of the surgery itself. The utility value of the subsequent cycles (progression of disease or disease-free) was based on the questionnaire completed at 6 months after surgery stratified for the health status at 6 months. We only included patients from this ongoing cohort study when they had returned the follow-up questionnaires after 1 and 6 months. Due to the limited number of observations per indication (LARC and LRRC), we did not stratify for LARC or LRRC, assuming similar QoL when having progression or being disease-free (Table 2). Furthermore, we did not stratify for navigation or standard surgery as we hypothesize that the effect of using navigation is found in the number of patients showing progression of disease as a consequence of a higher positive resection margin rate.

#### Intervention costs

For the costs of surgery, the formally average registered tariff (DRG) for open and laparoscopic low anterior resection (LAR) and abdominoperineal resection (APR) in the Netherlands were used. Open and laparoscopic APR and LRP showed the same tariff [21]. The additional costs for using the image-guided navigation system were estimated using a bottom-up costing methodology, taking into account additional activities, additional required time, and personnel. We assumed implementing navigation in an already existing hybrid OR and, for the base case, exclusive use of the navigation system for this indication (12%). This resulted in an additional cost of €3.388 per patient. Details on the calculation are described in Supplement 4.

#### State and transition costs

The health state costs and transition costs were based on the care delivered per state and transition. Care consumed per health state was based on the Dutch guideline on follow-up care for colorectal cancer [32]. Expert elicitation was used to estimate a weighted average of care used in case of an event (local recurrence, distant metastasis) for both LARC and LRRC, such as radiotherapy and chemoradiation. To calculate the transition costs, the identified consumed care was linked to tariffs for DRGs, health activities, and medications [22, 23, 33] (Table 2). Details on these costs are listed in Supplement 5.

### Model analysis and probabilistic sensitivity analysis

The models were built in Microsoft Excel, 2010. Costs were discounted at a rate of 4% and effects at a rate of 1.5% according to Dutch guidelines [18]. The primary outcome of the models was the ICER, which is calculated by dividing the incremental costs by the incremental QALYs. The involved experts of the NKI-AVL (TR, EK, GB) collaborated to validate the model, input parameters, and assumptions. Because this analysis evaluates an innovation early in its development process, the input parameters are subject to uncertainty. This uncertainty in the data and its effect on the ICER was evaluated using a probabilistic sensitivity analysis. Tables 1, 2 and 3 show the distributions surrounding the parameter values used in the Monte Carlo simulations (2000 random samples) for this analysis. The results of the probabilistic analysis are shown in a cost-effectiveness (CE-)plane. Furthermore, cost-effectiveness acceptability curves (CEAC) were generated, indicating the probability that an intervention is cost-effective, given a certain Willingness To Pay (WTP). The informal WTP ratio for diseases with a high symptom burden is €80.000 per QALY [34] in the Netherlands.

### Sensitivity analyses

In addition to the probabilistic sensitivity analysis, a deterministic one-way sensitivity analysis was performed, evaluating the influence of the uncertainty surrounding each of the input parameters. All parameters were varied over their upper and lower limits. The outcomes were plotted in a tornado diagram. Besides, two scenarios were evaluated: 1) Inclusion of construction costs for a hybrid OR to use the navigation system (in case a hospital does not have this yet), 2) Utilization of the navigation system was set at 50%, as it is assumed that the system is valuable in other indications as well. The input parameters for these scenarios are presented in Table 3 and detailed information is listed in Supplement 6. Finally, since the costs of the navigation system are still uncertain, a threshold analysis was performed assuming a WTP of €80.000 per QALY to identify the maximum incremental costs per patient [35].

**Table 3:** Parameters used in scenario analysis

	Combined LARC & LRRC	SE	Distribution	Source
**Scenario 1: Including the costs of constructing a hybrid OR when no hybrid OR is available in the hospital**				
Additional costs to use a hybrid OR and the C-arm CBCT in hybrid OR	€2.975			[25]; Supplement 4
Total costs of the addition of navigation	€6.363	€812	Gamma	
**Scenario 2: Using the navigation system for 50% instead of 12%**				
Navigation system	€1.027			Supplement 4
Addition of navigation costs per patient	€1.670	€213	Gamma	Expert; supplement 4

*SE* Standard error, *CBCT* Cone-Beam CT

### Value of information analysis

As this analysis was based on the first clinical data available for navigated surgery, the results are surrounded by a degree of uncertainty and therefore there is a chance that the ‘wrong’ policy decision is made. A value of information analysis provides insight in the worth of performing additional research, assuming that additional research would provide more certain estimates of the effects. The expected value of perfect information (EVPI), indicating the value of improved decision making by removing all uncertainty (i.e. by obtaining perfect information on all model parameters), was estimated [36]. Additionally, the expected value of partial perfect information (EVPPI) was calculated. This analysis shows the expected value of eliminating uncertainty on (a group of) specific input parameters. Both these analyses can thus be used to support decisions on further research in early stages of technology development. The EVPI was calculated by taking the difference between the expected net monetary benefit - obtained under perfect information - and the expected net monetary benefit obtained based on the current data. To evaluate the EVPI and EVPPI for the beneficial population, we used the yearly incidence numbers of LARC (*n* = 1384) and LRRC (*n* = 250) based on the Dutch situation [37, 38]. The population EVPI was evaluated for the coming 10 years and we discounted this population at a rate of 4%. The EVPPI was calculated for a willingness to pay threshold of €80.000.

## Results

### Base case results

Using the input parameters such as quality of life scores, risk of progression and chances of survival shown in Table 1 the incremental cost-effectiveness ratios for navigation use in LARC and LRRC were calculated. In this paragraph, the base case results – using the point estimates presented in Tables 1 and 2 – are presented. For LARC, we found 2.50 total Life Years (LY) after standard surgery versus 2.53 LY for navigated surgery. Total QALYs were 2.02 for standard and 2.05 for navigated surgery. Total costs for standard surgery were €23.238 compared to €26.379 for navigated surgery this amount includes the follow-up costs and costs for treating progression of disease. Dividing the incremental costs by the incremental effects resulted in an ICER of €136.604/QALY for LARC. Assuming a WTP ratio of €80.000 navigated surgery for LARC is judged not cost-effective.

For LRRC, we found 2.11 LYs after standard surgery compared to 2.17 LYs after navigated surgery. Total QALYs were 1.67 and 1.73 for standard and navigated surgery, respectively. Total costs of standard surgery were €25.862 and €28.719 for navigated surgery, including follow-up care and treating progression of disease. This early analysis resulted in an ICER of €51.802 per QALY gained (Table 4A). Assuming a WTP ratio of €80.000, navigated surgery for LRRC could be judged cost-effective.

**Table 4:** Deterministic outcomes of the cost-utility analysis on navigated surgery compared to standard surgery: base case and scenarios

**A. Base case results**							
	**Treatment costs**	**QALYs**	**LYs**	**iCosts**	**iQALYs**	**ICER**	**Conclusion**
**Base case results LARC**							
Navigated surgery	€26.379	2.05	2.53				Navigated surgery is more effective, more costly. Costs are above the WTP of €80.000/QALY
Standard surgery	€23.238	2.02	2.50				
				€3.141	0.02	€136.604	
**Base case results LRRC**							
Navigated surgery	€28.060	1.73	2.17				Navigated surgery is more effective, more costly. Costs are below the WTP of €80.000/QALY
Standard surgery	€25.164	1.67	2.11				
				€2.896	0.06	€52.510	
**B. Results from the scenario analysis**							
			**Intervention**		**ICER scenario**	**Conclusion scenario**	
Scenario 1: A hybrid OR has to be constructed before the navigation system can be used			LARC		€266.019	Navigated surgery is more effective, more costly. Costs are above the WTP of €80.000/QALY	
			LRRC		€106.458	Navigated surgery is more effective, more costly. Costs are above the WTP of €80.000/QALY	
Scenario 2: Increase in utilization of the navigation system to 50%			LARC		€61.817	Navigation is more effective, more costly. Costs are below the WTP of €80.000/QALY	
			LRRC		€21.334	Navigation is more effective, more costly. Costs are below the WTP of €80.000/QALY	
Combination of 1 and 2: increased use of the navigation system and including the costs of constructing a hybrid OR to use the navigation system#			LARC		€ 191.232	Navigated surgery is more effective, more costly. Costs are above the WTP of €80.000/QALY	
			LRRC		€ 75.282	Navigation is more effective, more costly. Costs are below the WTP of €80.000/QALY	

The WTP threshold used was €80.000. *QALYs* Quality of life years, *Lys* Life years, *iCosts* incremental costs, *iQALYs* incremental Quality of life years, *ICER* Incremental cost-effectiveness ratio, *WTP* Willingness To Pay threshold. # = total costs for the use of navigation including the hybrid OR costs assuming a use of 50% = €4.644,28

### Probabilistic sensitivity analysis

**Fig. 3:**
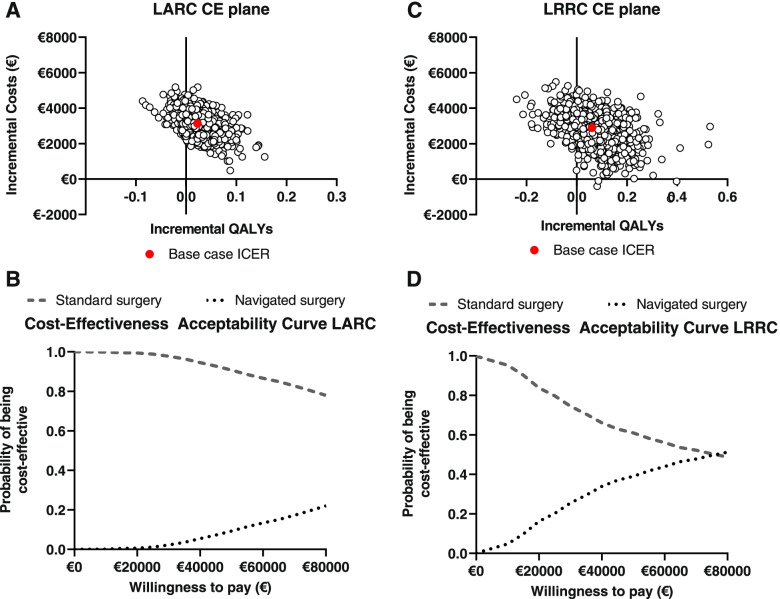
**A** and **C** show Cost-effectiveness planes for LARC (**A**) and LRRC (**C**) showing the incremental Quality Adjusted Life Years (QALYs) per incremental costs for navigated surgery versus standard surgery. The scatterplots show the mean differences in costs and outcomes from the data using 2000 bootstrap replicates. In both indications, most of the observations are in the North-East quadrant which indicates improved outcomes at higher costs. **B** and **D** show Cost-Effectiveness Acceptability Curves for LARC (**B**) and LRRC (**D**) presenting the probability of the cost-effectiveness of navigated surgery and standard surgery for a range of willingness to pay thresholds

The CE-plane in Fig. 3 shows that most observations for LARC (84%) indicated that navigated surgery resulted in better outcomes at higher costs. The Cost-Effectiveness Acceptability Curve (CEAC) shows that standard surgery in LARC has the highest probability of being cost-effective (78%) at a WTP of €80.000.

For LRRC, also most of the observations (79%) indicated improved outcomes at higher costs. The CE-plane shows more uncertainty compared to LARC, which corresponds to the smaller sample size in this study group. At a WTP threshold of €80.000, navigated surgery has a probability of 52% to be cost-effective compared to standard surgery for LRRC.

### Sensitivity analyses

**Fig. 4:**
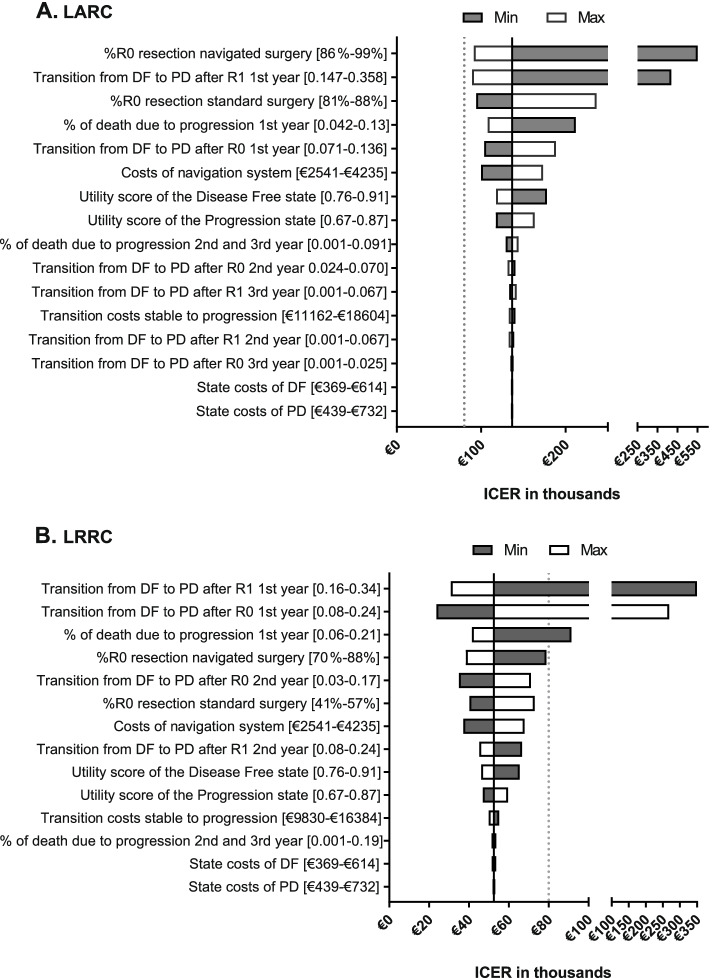
Sensitivity analyses. Tornado diagram showing the results of the one-way sensitivity analysis. **A** shows the results for the LARC group with a deterministic ICER of €136.604. **B** shows the results for the LRRC group with a deterministic ICER of €52.510. The scales of both figures are different and the gap on x-axis shows that a different scale is used after the gap. A dotted line is placed at the willingness to pay threshold of €80.000 which is used in the Netherlands. DF = disease free, PD = progression of disease, R0 = radical resection, R1 = a positive surgical margin

Figure 4 shows that the results are mostly influenced by the uncertainty surrounding the transition probabilities for the first year, the surgical margin rate, and the costs of the navigation system in both groups. For example, when the maximum value for the transition from disease-free to progression after an R0 resection was used - showing similar or even worse progression than after R1 resection - LRRC and LARC show ICERs around €200.000. Contrary, when the maximum value from disease-free to progression after an R1 resection was used, the ICERs for LARC and LRRC decreased substantially. For LRRC, this resulted for example in a QALY difference of 0.11 compared to 0.06 in the base case.

**Fig. 5:**
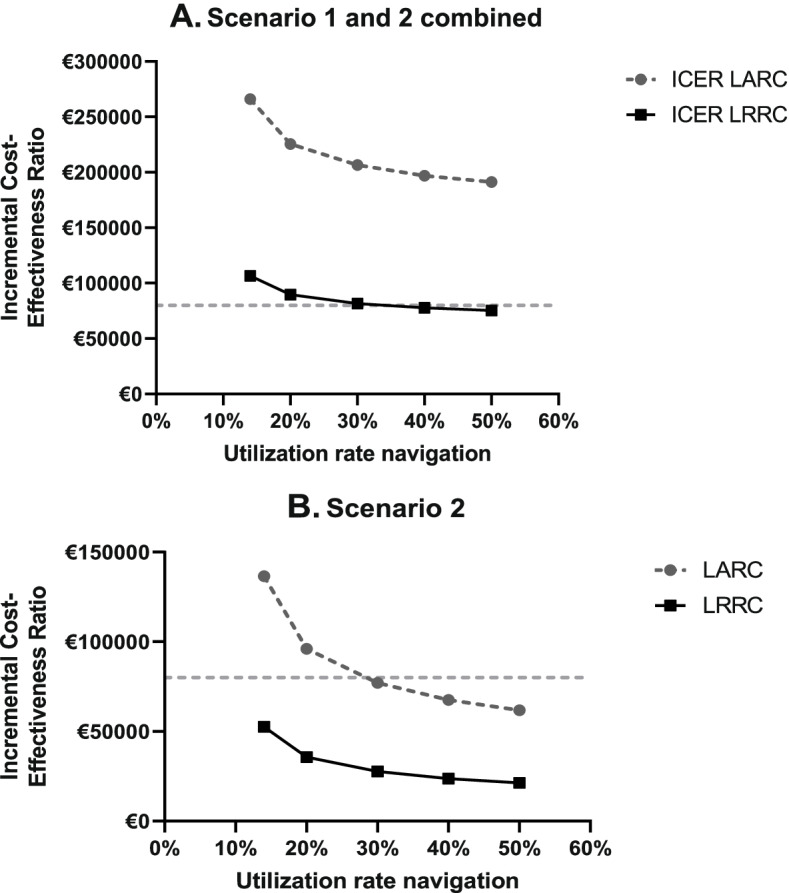
Graphical illustration of the scenario analysis. Shows the impact of varying the utilization rate of the navigation technology on the ICER. **A** shows the ICER for multiple utilization rates of navigation for the combination of scenario 1 and 2. Scenario 1 includes the construction costs for a hybrid OR when a hospital does not have this yet. In Scenario 2 the navigation system was used for 50%. **B** shows the ICER for multiple utilization rates of navigation of Scenario 2

Table 4B presents the results of the scenario analysis. When a hospital has to construct a hybrid OR before navigation can be used, navigated surgery is not cost-effective in LARC or LRRC (scenario 1). Increasing the utilization of the navigation system (scenario 2) results in navigated surgery being cost-effective at a WTP threshold of €80.000 for LARC. For LRRC, navigated surgery is then cost-effective at most commonly used WTP thresholds. Since this is a realistic scenario, supplement 7 shows the probabilistic results for this scenario, showing that navigated surgery has a probability of 67% to be cost-effective for LRRC patients. Figure 5 shows the effect of various utilization ratios on the ICER for scenario 2 and the combination of Scenario 1 and 2.

Based on the threshold analysis, we found that the navigation system may have a maximum cost per patient of €1.839 in LARC and €4.412 in LRRC.

### Value of information analysis

**Fig. 6:**
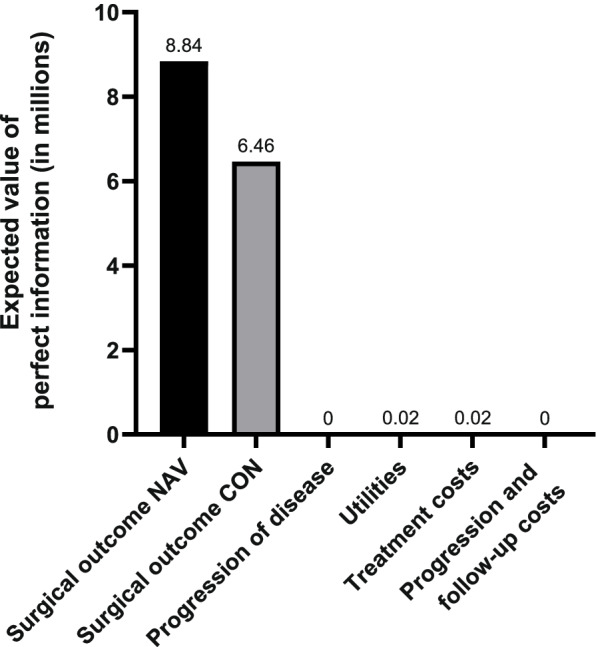
The expected value of perfect information for parameter groups for LRRC

The EVPI was almost €3.7 M in LRRC, which indicates the value of reducing the risk of making the wrong decision (e.g. reimbursing navigated surgery when actually not cost-effective) by performing further research (Supplement 8). The results of the EVPPI are presented in Fig. 6. This graph shows that obtaining more certain estimates on having a positive or negative resection margin after standard- and navigated surgery would be most valuable. Other valuable topics for further research were the utility values and treatment costs (including the navigation costs). Since standard surgery was preferred at a WTP threshold of €80.000 in LARC, estimating the EVPI was not considered relevant for LARC.

## Discussion

This early evaluation indicates that navigated surgery is expected to be cost-effective in LRRC patients (ICER: €52.510). Furthermore, it has the potential to become cost-effective for LARC when the costs of the navigation system would decrease by for example increased use or price negotiations. These results are in line with the promising clinical results of Kok et al. [12].

Based on Fig. 4, a strong relationship between an R0 resection and a reduced risk of recurrence seems crucial for navigated surgery to become cost-effective. Although we concluded that navigation is cost-effective in LRRC, our data for LRRC showed no clear relationship between an R0 resection and a reduced risk of progression, e.g. reflected by the limited QALY gain found. Based on the significantly higher chance to achieve an R0 resection (79% vs 49% (*p* = 0.047)) [12] we expected a larger QALY difference. In a best-case situation, having a lower risk of progression with an R0 resection, the ICER could drop to €23.648 (Fig. 3). Based on the current evidence base [16, 39, 40], it could be expected that a stronger relation between R0 and reduced risk on progression is found when the analysis is based on a larger dataset, and progression of disease is stratified in local recurrence and distant metastasis. This would result in a higher chance for navigated surgery to become cost-effective. It should, however, be noted that resection margin status is also influenced by tumor biology.

Although a strong relationship between an R0 resection and a reduced risk of progression was found in LARC, navigated surgery was not cost-effective in the base case analysis, since the difference in having an R0 resection between standard and navigated surgery was small [12]. Identification of clinical subtypes that would especially benefit from navigated surgery would be of interest to become cost-effective in LARC.

The navigation system costs seem another crucial aspect that influenced the results. One could consider pricing the navigation system related to its cost-effectiveness, as in value-based pricing. The threshold analysis showed that the maximum per patient cost may be €1.839 in LARC and €4.412 in LRRC. When the navigation system is used in multiple indications, the value base in each of these indications should be taken into account.

A final important finding is that, when investing in a navigation system, users should aim for optimal capacity use. For instance by organizing centralization of care or identification of multiple indications where navigation could be of added value (Table 4). This is especially the case when a hospital has to invest in a hybrid OR. Currently, the use of navigation is piloted in multiple oncologic indications [19, 41, 42], which could facilitate the future adoption of navigated surgery.

This study presents results from the first cost-effectiveness analysis for navigated surgery based on the first clinical data available. The results could inform its further development and the start of subsequent clinical or pilot studies. Further strengths include, (i) the inclusion of tunnel states to incorporate time-to-event information, and (ii) the utility values that were based on prospective data from a relatively large (*n* = 63) and similar patient group, that showed utility values that were in line with literature [43].

This study has several limitations that should be acknowledged. In general, early cost-effectiveness analyses are associated with uncertainty in the input parameters, for example, because of small sample sizes and suboptimal study designs. Therefore, the outcomes could be debatable. This is also shown by the CEAC (using the uncertainty surrounding the input parameters) for LRRC, showing a probability of only 52% that navigated surgery is cost-effective at a WTP threshold of €80.000. The value of information analysis shows that it is worthwhile to perform further research especially focusing on the risk of having an R0 or R1 resection after navigated- and standard surgery. Furthermore, based on the available dataset it was not possible to test the influence of a longer follow-up period.

More specifically, our analysis is limited because we did not incorporate patient characteristics (e.g. tumor stage, tumor location) since treatment history could affect margin status and progression of disease [44]. Besides, by not stratifying for local recurrence and distant metastasis, evaluation of the relationship between R0 and a reduced risk of progression was challenging, because achieving an R0 resection has a limited influence on reducing the risk of metastases.

Related to the utility values, the utility-scores for LARC and LRRC were assumed to be equal, although LRRC patients are expected to receive multiple chemotherapy lines which is expected to result in lower utility values [45]. Furthermore, we should note that we could not include all patients included in the ongoing trial because of missing data. Another limitation is that we used the 6-month questionnaire to base our utility value on for the disease-free and progression state, which is likely to overestimate the utility-score since patients experience more complaints as the disease progresses. As the utility scores show a large impact on the cost-effectiveness results, utility values for 1 and 2 years after surgery should be incorporated. Furthermore, as curative surgery (R0 resection) is expected to decrease pain complaints, we expect to underestimate the QoL benefit of navigated surgery leading to rather negative ICER estimates. Another issue is related to the costs used in the analysis. The costs were calculated from a Dutch healthcare perspective while the costs of the navigation system were based on list prices and expert elicitation. Finally, since the navigation system is a new surgical tool, a learning curve may be present which potentially underestimates the performance of navigated surgery.

This analysis should be seen as a first step in evaluating the added value of navigated surgery for LRRC and LARC. Although there is a tendency in surgery to not formally evaluate incremental improvements in technology, we recommend comparing navigated and standard surgery prospectively, preferably multi-center, in terms of resection margin rate (R0, R1, and R2), complication rate, QoL and utility values. Based on this data, the cost-effectiveness should be updated using the large (inter) national studies presenting survival after R0 and R1/R2 margins [15, 16, 39, 40] to inform adoption and reimbursement decisions, and validate the results of this study. Furthermore, we suggest validating the mapping study of Wong et al. when QoL and utilities are measured at several moments in time since the EQ5D-5L seems not sensitive to capture CRC specific complaints [46]. Subsequently, to inform decision-makers with the best available evidence, also on potential unforeseen effects e.g. learning curve, the cost-effectiveness analysis should be updated (iterative approach [47]) when more robust survival data is available.

## Conclusion

Based on this early cost-effectiveness analysis, navigated surgery is expected to be cost-effective in LRRC patients and it has the potential to become cost-effective for LARC patients. Further research, preferably prospective studies, is necessary to validate these results and conclude on routine use of navigation and reimbursement of navigated surgery. Since the navigation system seems to be associated with high costs per patient, it is crucial to, when hospitals invest in such an innovative medical device, use it optimally (centralization of care) and seek other indications where it could be of additional value.

## Supplementary Information

**Additional file 1.: Supplement 1.** Patient characteristics of patients included in the study of Kok et al. 2020. **Supplement 2.** Characteristics of patients included in the prospective study evaluating quality of life. **Supplement 3.** Overview of sources for input of the model. **Supplement 4.** Details on the additional costs for using the navigation system during surgery. **Supplement 5.** Details of state costs. **Supplement 6.** Detailed information on the scenario input parameters. **Supplement 7.** Probabilistic results for LARC and LRRC when Scenario 2 is present. **Supplement 8.** Graphical visualization of Expected Value of Perfect Information.

## Data Availability

The datasets used and/or analysed during the current study are stored in the repository of the Netherlands Cancer Institute and are available from the corresponding author on reasonable request. All input parameters are presented in the manuscript or supplementary files and can be used to reproduce our analysis and results.

## Abbreviations

APR: Abdominoperineal resection
CE: Cost-effectiveness
CEAC: Cost-effectiveness acceptability curves
CRC: Colorectal cancer
DRG: Diagnosis-related group
EVPI: Expected value of perfect information
ICER: Incremental cost-effectiveness ratio
LAR: Los anterior resection
LARC: Locally advanced rectal cancer
LRRC: Locally recurrent rectal cancer
LY: Life-years
NKI – AVL: Netherlands cancer institute – Antoni van leeuwenhoek
QALY: Quality adjusted life years
QoL: Quality of Life
R0: Negative resection margin
R1: Positive resection margin
WTP: Willingness to pay
