# Supplementary material for: Image-guided navigation for locally advanced primary and locally recurrent rectal cancer: evaluation of its early cost-effectiveness
Source: BMC Cancer. 2022 May 6;22:504. doi: 10.1186/s12885-022-09561-w (PMC9074374; doi:10.1186/s12885-022-09561-w)
Supplement: Supplementary file 1 — Additional file 1.: Supplement 1. Patient characteristics of patients included in the study of Kok et al. 2020. Supplement 2. Characteristics of patients included in the prospective study evaluating quality of life. Supplement 3. Overview of sources for input of the model. Supplement 4. Details on the additional costs for using the navigation system during surgery. Supplement 5. Details of state costs. Supplement 6. Detailed information on the scenario input parameters. Supplement 7. Probabilistic results for LARC and LRRC when Scenario 2 is present. Supplement 8. Graphical visualization of Expected Value of Perfect Information. [file 12885_2022_9561_MOESM1_ESM.docx]

# Supplements

## Supplement 1 – Patient characteristics of patients included in the study of Kok et al. 2020

|  | **Navigation group (A)** | **Control group (B)** | **P-value** |
| --- | --- | --- | --- |
| Locally advanced primary rectal cancer | **N = 14** | **N = 101** |  |
| Sex   - Male - Female | 12 (85.7)  2 (14.3) | 73 (72.3)  28 (27.7) | 0.351 |
| Age at start treatment (median, years) | 58.0 (35 – 71) | 61.0 (25 - 82) | 0.486 |
| Clinical tumor and nodal stage primary tumor   - T3N0-2 MRF+ - T4N0-2 MRF+ | 7 (50)  7 (50) | 62 (61.4)  39 (38.6) | 0.562 |
| Distant metastases   - Present - Absent | 3 (21.4)  11 (78.6) | 24 (23.8)  77 (78.6) | 1.00 |
| Primary tumors location from anorectal verge   - Low (0 - 5 cm) - Middle (5 – 10 cm) - High (10 – 15 cm) | 10 (71.4)  3 (21.4)  1 (7.1) | 52 (51.5)  31 (30.7)  18 (17.8) | 0.461 |
| Neoadjuvant treatment primary tumor   - None - Short course radiotherapy (5 x 5 Gy) - Chemoradiation - 5x5 Gy + chemotherapy - Chemoradiation + chemotherapy | 0 (0)  0 (0)  8 (57.2)  5 (35.7)  1 (7.1) | 1 (1.0)  3 (3.0)  80 (79.2)  16 (15.8)  1 (1.0) | 0.130 |
| Type of surgery   - Open APR - Lap. APR - Open LAR - Lap. LAR - Exenteration | 2 (14.3)  0 (0)  12 (78.6)  1 (7.1)  0 (0) | 38 (37.6)  3 (3.0)  35 (34.7)  10 (9.9)  15 (14.9) | 0.103 |
| Pathological outcome |  |  | 0.390 |
| ypT0N0  ypT2N0-2  ypT3N0  ypT3N1  ypT3N2  ypT3N04  ypT4N1  ypT4N2 | 2 (14.3)  2 (14.3)  2 (14.3)  3 (21.4)  4 (28.6)  0 (0)  1 (7.1)  0 (0) | 5 (5.0)  8 (7.9)  35 (34.7)  14 (13.9)  28 (27.7)  7 (6.9)  1 (3.0)  1 (1.0) |  |
| Recurrent rectal cancer | **N = 19** | **N = 41** |  |
| Sex   - Male - Female | 11 (57.9)  8 (42.1) | 22 (53.7)  19 (46.3) | 0.788 |
| Age at start treatment (median, years) | 61.5 (52 – 78) | 67.0 (41 – 82) | 0.079 |
| Recurrent tumor location   - Pelvic wall / presacral - Staple line recurrence | 14 (73.7)  5 (26.3) | 26 (63.9)  15 (36.6) | 0.560 |
| Neoadjuvant treatment recurrent rectal cancer   - None - Short course radiotherapy - Chemotherapy - Chemoradiation - Chemoradiation + chemotherapy | 0 (0)  0 (0)  1 (5.3)  11 (57.9)  7 (36.8) | 5 (12.2)  2 (4.9)  2 (4.9)  29 (70.7)  3 (7.3) | 0.061 |
| Type of surgery   - Open APR - Open LAR - Exenteration - Local resection | 3 (15.8)  4 (21.1)  8 (42.1)  4 (20.0) | 19 (46.3)  6 (14.6)  14 (34.2)  2 (4.9) | 0.100 |

APR = Abdominal Perineal Resection, LAR = Lower Anterior Resection; similar to table 1 of Kok et al. 2020 in JAMA network open.

## Supplement 2 – Characteristics of patients included in the Quality of Life input

|  | **Respondents completed the first-month questionnaire** | **Respondents completed the 6-month questionnaire** | |
| --- | --- | --- | --- |
|  |  | **Disease-free at 6 months** | **Progressive disease at 6 months** |
|  | **N = 63** | **N = 44** | **N =14** |
| Sex   - Male - Female | 47 (74.6%)  16 (25.4%) | 28 (63.6%)  16 (36.4%) | 10 (71.4%)  4 (28.6%) |
| Age at start treatment (median, years, range) | 62.0 (35-82) | 64.50 (47-79) | 67.50 (35-82) |
| Clinical tumor and nodal stage primary tumor   - Recurrence - T2N0-2 - T3N0-2 - T4N0-2 | 18 (28.6%)  6 (9.5%)  30 (47.6%)  9 (14.3%) | 12 (27.3%)  6 (13.6%)  21 (47.7%)  5 (11.4%) | 5 (35.7%)  0  5 (34.7%)  4 (28.6%) |
| Type of tumor  Primary rectal cancer (LARC)  Recurrent rectal cancer (LRRC) | 48 (76.2%)  18 (28.6%) | 32 (72.7%)  12 (27.3%) | 9 (64.3%)  5 (35.7%) |
| Distant metastases  Present  Absent | 15 (23.8%)  48 (76.2%) | 4 (9.1%)  40 (90.9%) | 8 (57.1%)  6 (42.9%) |
| Primary tumors location from anorectal verge   - Low (0 - 5 cm) - Middle (5 – 10 cm) - High (10 – 15 cm) | 38 (60.3%)  22 (34.9%)  3 (4.8%) | 26 (59.1%)  16 (36.4%)  2 (4.5%) | 5 (35.7%)  7 (50.0%)  2 (14.3%) |
| Neoadjuvant treatment primary tumor   - None - Short course radiotherapy (5 x 5 Gy) - Chemoradiation - 5x5 Gy + chemotherapy - Chemoradiation + chemotherapy - Chemotherapy | 6 (9.5%)  3 (4.8%)  36 (57.1%)  11 (17.5%)  6 (9.5%)  1 (1.6%) | 3 (6.8%)  3 (6.8%)  31 (70.5%)  3 (6.8%)  4 (9.1%)  0 | 1 (7.1%)  0  4 (28.6%)  6 (42.9%)  2 (14.3%)  1 (7.1%) |
| Type of surgery   - Open APR - Lap. APR - Open LAR - Lap. LAR - Exenteration - Local resection - Hipec | 15 (23.8%)  13 (20.6%)  14 (22.2%)  11 (17.5%)  7 (11.1%)  2 (3.2%)  1 (1.6%) | 9 (20.5%)  10 (22.7%)  8 (18.2%)  8 (18.2%)  6 (13.6%)  2 (4.5%)  1 (2.3%) | 3 (21.4%)  1 (7.1%)  5 (35.7%)  2 (14.3%)  3 (21.4%)  0  0 |
| Navigation  Yes  No | 36 (51.7%)  27 (42.9%) | 18 (40.9%)  26 (59.1%) | 6 (42.9%)  8 (57.1%) |

## Supplement 3 – Overview of sources for input of the model


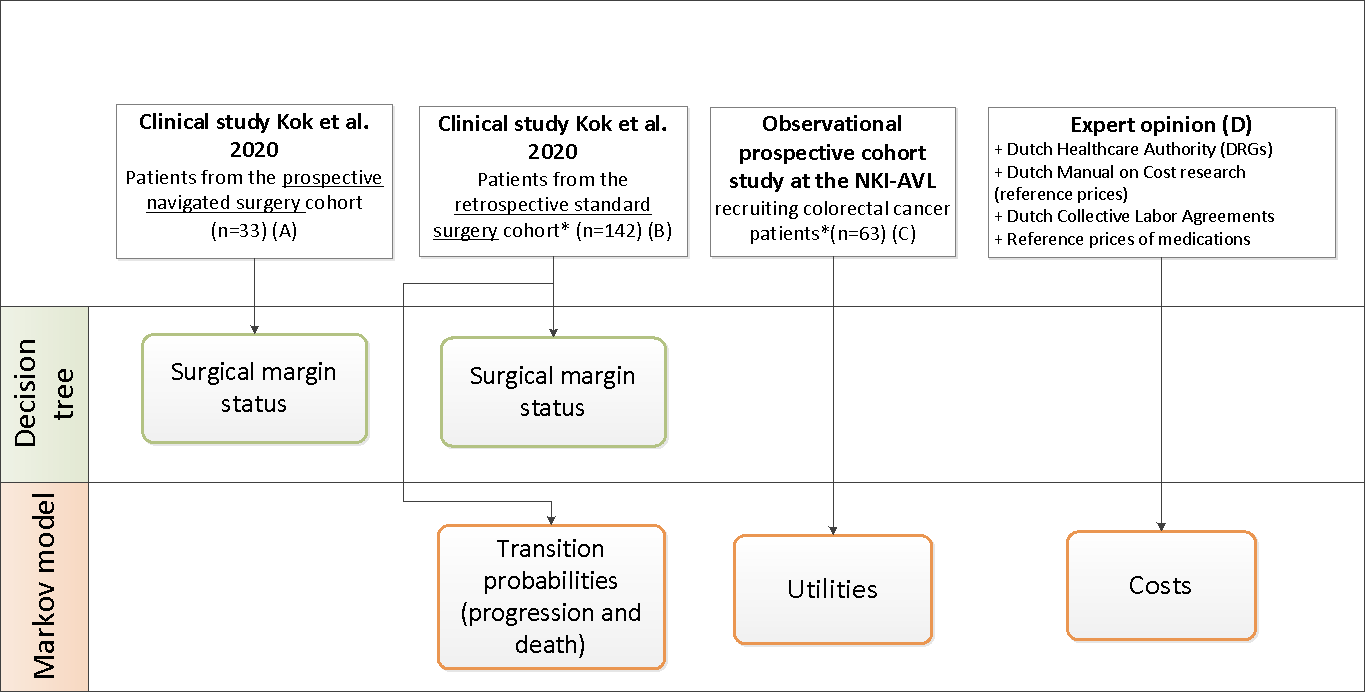


Caption Supplement 3: Schematic overview of studies used for input of the model; * same inclusion criteria

## Supplement 4 - Details on the additional costs for using the navigation system during surgery

Prior to navigated surgery, a CT scan is performed to build the 3D anatomical model. The intervention and personnel costs for building the 3D anatomical model result in €269(1–3). For the attendance of a technician during the surgery €197 was charged, calculated by taking the average duration of the procedure (5,73h) multiplied with the annual loaded salary retrieved from Collective Labor Agreements(1,2). The costs of the navigation system, as still in development are yet unknown. Therefore, an average was based on list prices of comparable systems recently launched or planned to launch for example by Intuitive and Auris Health. Assuming a lifetime of 10 years, annual interest percentage of 4.2%(4), the utilization rate of the navigation system at the NKI-AVL (12%), service costs (10%) and disposable costs (€500), resulted in an expected costs of €2.745 per patient. Overhead (38%) was calculated over the costs of the additional steps and personnel costs (€177)(1). We did not include the additional preparation time of less than 20 minutes as it is assumed to be balanced out by the reduced duration to find the tumor(5). In total, the use of the navigation system during surgery resulted in €3.388 per patient (Table 2). In this estimation, we assume that a hospital has a hybrid OR with a Cone-beam CT scanner system.

## Supplement 5 - Details of state costs

| **State** **costs (LARC & LRRC)** | | | | | | | |
| --- | --- | --- | --- | --- | --- | --- | --- |
|  | | | | Times per year* | Costs per activity | Costs per year | Source |
| **Disease free state** | | | | | | | |
|  | CEA test | | | 4 | 7,54 | € 30,16 | (6) |
|  | face-to-face consultation | | | 2 | 168,61 | € 337,21 | (4) |
|  | CT abdomen + CT rectum | | | 2 | 375,04 | € 750,08 | (6) |
|  | Consultation over phone | | | 2 | 17,69 | € 35,38 | (4) |
|  | MRI-scan | | | 0,5 | 316,95 | € 158,47 | (6) |
|  | PET/CT-scan | | | 0,5 | 1069,76 | € 534,88 | (6) |
|  | Colonoscopy | | | 0,5 | 240,24 | € 120,12 | (6) |
| Follow-up care per year | | | |  |  | € 1.966,31 |  |
| Follow-up care per cycle (3 months) | | | |  |  | € 491,58 |  |
| **Progression of disease state** | | | | | | | |
|  | CEA | | | 4 | 7,54 | € 30,16 | (6) |
|  | face-to-face consultation | | | 2 | 168,61 | € 337,21 | (4) |
|  | CT abdomen + CT rectum | | | 3 | 375,04 | € 1125,12 | (6) |
|  | Consultation over phone | | | 2 | 17,69 | € 35,38 | (4) |
|  | MRI-scan | | | 0,5 | 316,95 | € 158,475 | (6) |
|  | PET/CT-scan | | | 0,5 | 1069,76 | € 534,88 | (6) |
|  | Colonoscopy | | | 0,5 | 240,24 | € 120,12 | (6) |
| Follow-up care per year | | | |  |  | € 2.341,35 |  |
| Follow-up care per cycle (3 months) | | | |  |  | € 585,34 |  |
|  | |  | |  |  |  |  |
| **Costs of progression** | | | | % of patients | Costs per activity | Costs * % | Source |
| Local recurrence (LARC) | | | |  |  |  |  |
|  | Surgery | | | 50% | €10.040,00 | € 5.020,00 | (7) |
|  | Radiotherapy (3D) | | | 5% | €6.865,00 | € 343,25 | (7) |
|  | Chemoradiotherapy (CRT: 25 x 2 Gy met capecitabine) | | | 90% | €7.592,50 | €6.833,25 | (7–9) |
|  | Chemotherapy (6 cycles of capox) | | | 20% | € 7.590,36 | €1.518,07 | (10,11) |
|  | palliative chemotherapy | | | 25% | € 7.590,36 | € 1.897,59 | (10) |
| Total treatment costs: | | | |  |  | € 15.612,16 |  |
| Local recurrence (LRRC) | | | | | | | |
|  | Surgery | | | 25% | € 10.040,00 | € 2.510,00 | (7) |
|  | Radiotherapy (extern) | | | 5% | € 6.865,00 | € 343,25 | (7) |
|  | Chemoradiotherapy (same as LARC) | | | 20% | € 7.547,50 | € 1.509,50 | (7,10,11) |
|  | Chemotherapy (same as LARC) | | | 20% | € 7.590,36 | € 1.518,07 | (10,11) |
|  | Palliative chemotherapy | | | 80% | € 7.590,36 | € 6.072,29 | (10) |
| Total treatment costs: | | | |  |  | € 11.953,11 |  |
| Distant metastasis | | | | | | | |
|  | Radiotherapy (extern) | | | 10% | € 7.485,00 | € 748,50 | (7) |
|  | Chemoradiotherapy | | | 10% | € 8.000,00 | € 800,00 | (7,10,11) |
|  | Chemotherapy | | | 90% | € 6.813,24 | € 6.131,92 | (7,10,11) |
|  | Surgery (liver/lung/lymph) | | | 30% | € 15.360,00 | € 4.608,00 | (7) |
|  | RFA/MWA | | | 10% | € 15.360,00 | € 1.536,00 | (7) |
|  | Immunotherapy (nivolumab) | | | 3% | € 11.006,58 | € 330,20 | (10) |
|  | HIPEC | | | 5% | € 10.930,00 | € 546,50 | (7) |
| Total treatment costs: | | | |  |  | € 14.701,11 |  |
|  | | |  |  |  |  |  |
|  | | | | % of patients with LR | % of patients with DM | Total treatment costs |  |
| Transitions costs of progression LARC | | | | 20% | 80% | € 14,883.32 | [B] |
| Transitions costs of progression LRRC | | | | 42% | 58% | € 13,107.27 | [B] |

*= for 5 years. LR = local recurrence, DM = distant metastasis

## Supplement 6 – Detailed information on the scenario input parameters

For scenario 1, we evaluated the costs when a hybrid OR needs to be constructed and a fixed C-arm CBCT is used. Based on a Dutch study evaluating the additional costs of the hybrid OR compared to a conventional OR, 3,43 euros per minute are accounted for general additional inventory and higher construction costs. The C-arm CBCT adds on average 5,22 euros per minute. For the average duration of the surgery per patient (5,73h (range of 3,08h-10,4h)), the use of the hybrid operating room including a C-arm costs €2.975 based on a recent evaluation of the costs of conventional and hybrid operating rooms(12).

For scenario 2, we evaluated the costs of the navigation system when its use increases from 12% to 50%. This results in a decreased cost of €1.027 to use the navigation system solely instead of € 2.745 (Table 3). Resulting in a total cost of €1.670 including the additional steps to use the navigation during a procedure.

## Supplement 7 - Probabilistic results for LARC and LRRC when Scenario 2 is present


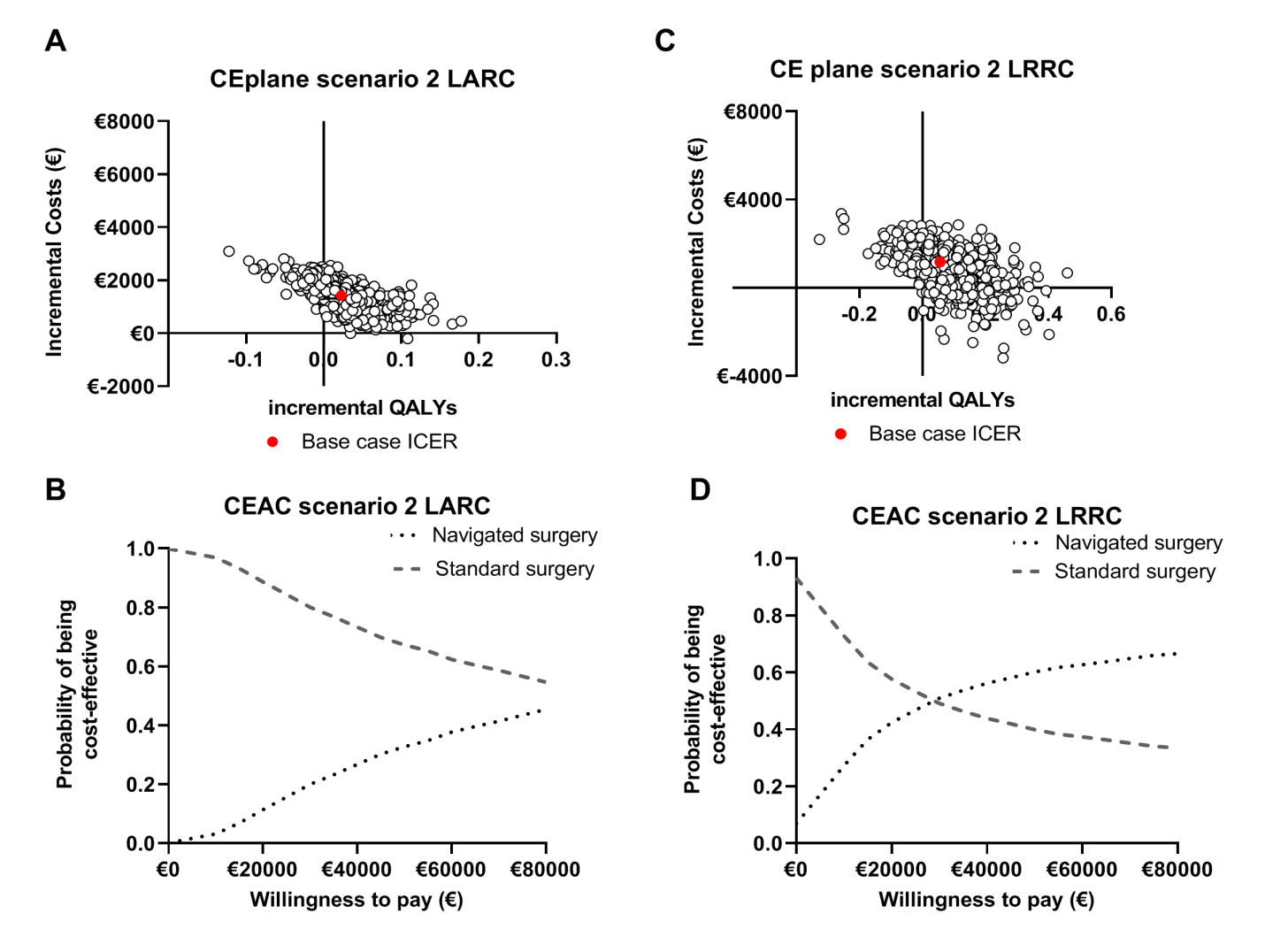
Caption Supplement 7; Shows the probabilistic sensitivity analysis results when the navigation system is used more often (Scenario 2). A and C show Cost-effectiveness planes for LARC (A) and LRRC (C) with the decreased costs of the navigation system showing the incremental Quality Adjusted Life Years (QALYs) per incremental costs for navigated surgery versus standard surgery. The scatterplots show the mean differences in costs and outcomes from the data using 2000 bootstrap replicates. B and D show Cost-Effectiveness Acceptability Curves for LARC (B) and LRRC (D) presenting the probability of the cost-effectiveness of navigated surgery and standard surgery for a range of willingness to pay thresholds.

## Supplement 8 – Graphical visualization of Expected Value of Perfect Information


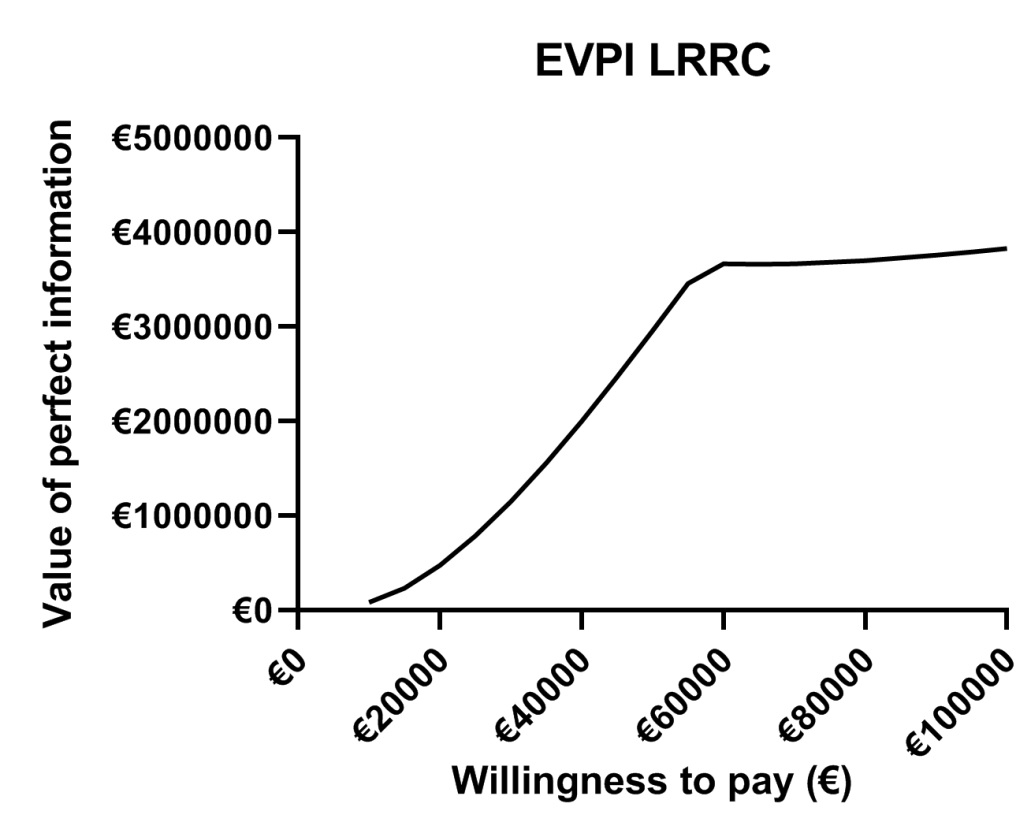


The line presents the expected value of perfect information. The highest value surrounding the willingness to pay threshold of €60.000 is 3.7milion indicating that 3.7 million is needed to obtain perfect information on whether navigated surgery is cost-effective at a threshold of €60.000.

**References used in supplementary material**

1. Hakkaart-van Roijen L, van der Linden N, Bouwmans C, Kanters T, Swan Tan S. Manual for cost research: methods and standard cost prices for economic evaluations in health care. Diemen; 2015.

2. Nederlandse Vereniging van Ziekenhuizen (NVZ). Salarisschalen Cao Ziekenhuizen (2018) (Dutch collective labor agreement). 2018.

3. Dutch Healthcare Authority (NZa). DBC product finder for tariffs [Internet]. 2019 [cited 2017 Feb 2]. Available from: http://dbc-zorgproducten-tarieven.nza.nl

4. Zorginstituut Nederland (Dutch institute of healthcare). Richtlijn voor het uitvoeren van economische evaluaties in de gezondheidszorg. 2016.

5. Nijkamp J, Kuhlmann KFD, Ivashchenko O, Pouw B, Hoetjes N, Lindenberg MA, et al. Prospective study on image-guided navigation surgery for pelvic malignancies. Journal of Surgical Oncology. 2019 Mar;119(4):510–7.

6. Nederlandse Zorgautoriteit. NZa zorgproductapplicatie.

7. Nederlandse Zorgautoriteit. Open data van de Nederlandse Zorgautoriteit [Internet]. Nederlandse Zorgautoriteit [Internet]. 2019. Available from: https://www.opendisdata.nl/

8. Zorginstituut Nederland. Farmaceutisch Kompas.

9. Zorginstituut Nederland. Medicijnkosten.nl. 2019.

10. Zorginstituut Nederland. Medicijnkosten.nl. 2019.

11. Zorginstituut Nederland. Farmaceutisch Kompas.

12. Patel S, Lindenberg M, Rovers MM, van Harten WH, Ruers TJM, Poot L, et al. Understanding the Costs of Surgery: A Bottom-Up Cost Analysis of Both a Hybrid Operating Room and Conventional Operating Room. International Journal of Health Policy and Management [Internet]. 2020 Jul 27; Available from: https://www.ijhpm.com/article_3870.html
